# Mixed Germ Cell Tumor of Testis with Isolated Scapular Metastasis: A Case Report and Review of the Literature

**DOI:** 10.1155/2015/205297

**Published:** 2015-08-16

**Authors:** Dipti Rani Samanta, Chaitali Bose, Roopesh Krishnappa, Saumyaranjan Mishra, Sulagna Mohanty, Ashish Upadhyay, Surendra Nath Senapati

**Affiliations:** ^1^Medical Oncology, A. H. Regional Cancer Centre, Odisha 753007, India; ^2^Radiation Oncology, A. H. Regional Cancer Centre, Odisha 753007, India; ^3^Radiation Oncology, Apollo Hospital, Hyderabad, Andhra Pradesh, India

## Abstract

Bone metastasis is a rare entity in germ cell tumor of testis and is a poor prognostic site. It is usually associated with synchronous metastasis at other sites. Till now very few cases of isolated bone metastasis of germ cell tumor of testis have been reported but none have reported scapular metastasis. We are reporting a case of nonseminomatous germ cell tumor of right testis that was operated eight months ago and now presented with isolated scapular metastasis. Histopathology of the scapular tissue revealed rhabdomyosarcoma or poorly differentiated synovial sarcoma. Immunohistochemistry with serum markers concluded it to be metastatic germ cell tumor. To the best of our knowledge this is the first reported case of scapular metastasis of testicular germ cell tumor. This case is being reported here due to dilemmatic way of presentation and also to emphasize that histopathology may sometimes misguide and immunohistochemistry is necessary in such cases.

## 1. Introduction

Testicular tumor accounts for approximately 1% of all the tumors in male. It is the most common solid malignancy among the males in the age group of 15 to 35 years [1]. Mixed germ cell tumors are the second most common testicular germ cell tumor accounting for 40–50% of all primary germ cell tumors. Irrespective of their histology, testicular tumor usually metastasizes to retroperitoneal lymph node. In advanced stage there is also hematogenous metastasis to lung, liver, brain, and less commonly other organs of body. Bone metastasis is an uncommon entity. Nonpulmonary visceral metastasis is considered as a poor prognostic feature. Bone metastasis classifies patient into poor (nonseminomatous) or intermediate (seminomatous) prognostic group [2]. Here is a case of mixed germ cell tumor of right testis with scapular metastasis. Although histopathology report of scapular biopsy simulated rhabdomyosarcoma or poorly differentiated synovial sarcoma, immunohistochemistry and serum markers confirmed it as metastatic mixed germ cell tumor. This case is reported because of rarity of scapular metastasis from mixed germ cell tumor of testis and its confusing way of presentation.

## 2. Case Report

**Figure 1:**
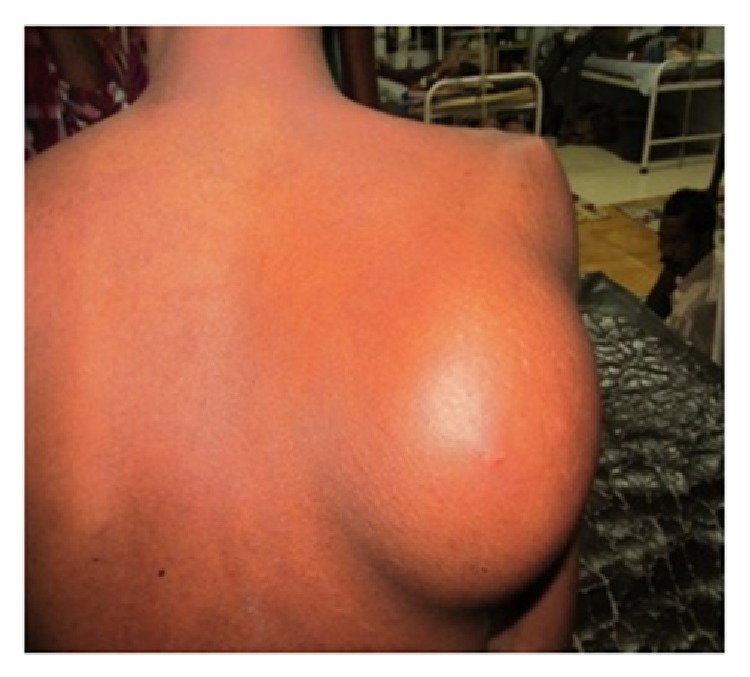
Clinical photograph of showing scapular swelling before chemotherapy.

A 22-year-old male presented with progressive swelling over right scapular region of 8-month duration. He had undergone orchidectomy of the right testis at a periphery hospital 1 year ago. The treating surgeon had not sent the tissue for histopathology study, as he had no oncology experience. On local examination, a swelling of size 15 × 12 cm was found over right scapular region which was hard, smooth, and fixed to scapula (Figure 1). The rest of the physical examination was normal except right scrotum which was empty due to previous orchidectomy.

**Figure 2:**
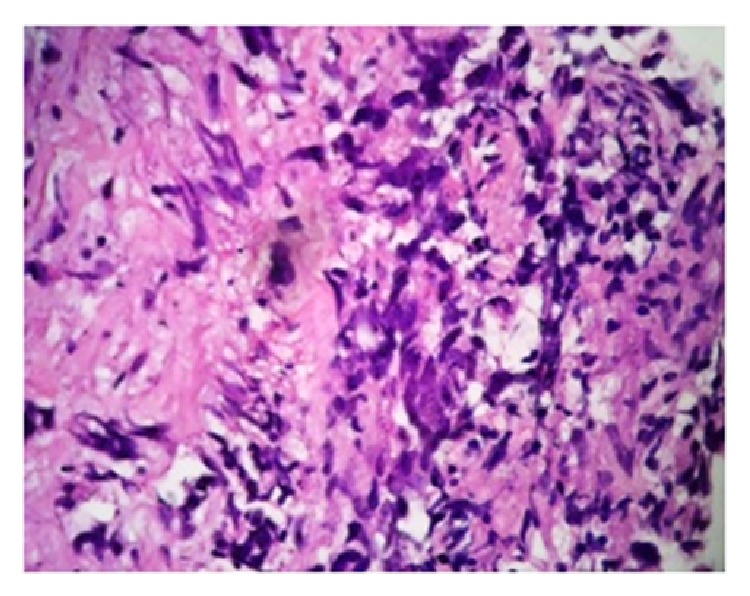
Photomicrograph of biopsy taken from scapular swelling showing ovoid shaped dark cells can be noticed which gives sarcomatous picture.

**Figure 3:**
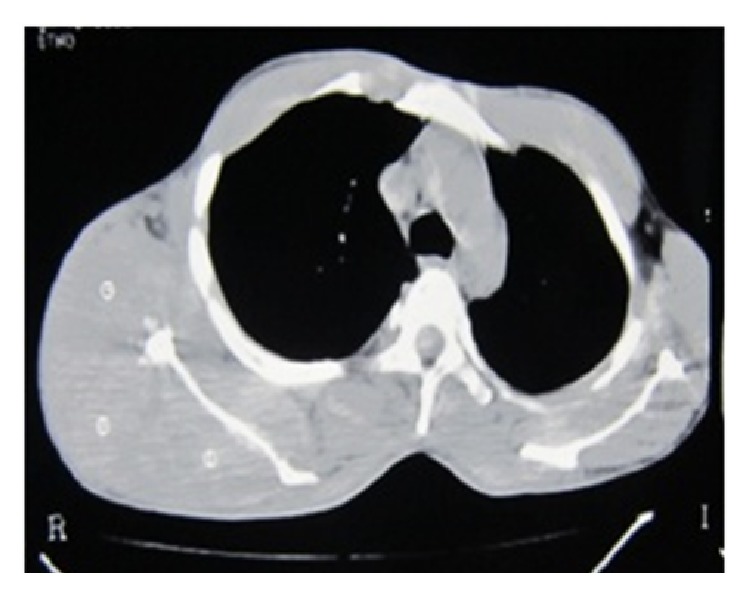
Contrast enhanced CT scan showing huge scapular swelling.

**Figure 4:**
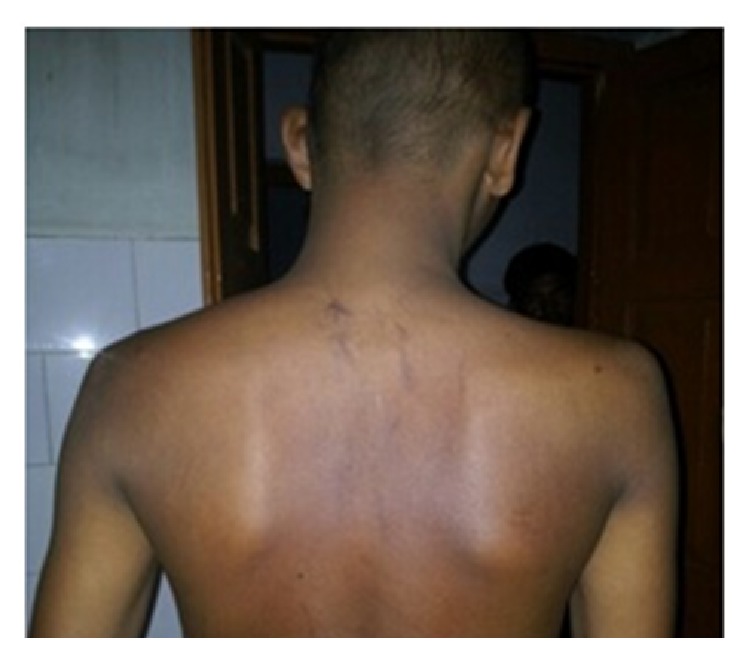
Clinical photograph showing reduced swelling after 3 cycles of chemotherapy.

Fine needle aspiration cytology of scapular swelling was suggestive of extra gonadal germ cell tumor. However biopsy of the swelling revealed striated muscle bundles and fibrocollagenous stroma with lobules of round to ovoid dark cells with scanty cytoplasm suggestive of either alveolar rhabdomyosarcoma or poorly differentiated synovial sarcoma (Figure 2). Computed tomography of thorax showed an enhancing mass over right scapular region of size 17.1 × 12.5 cm invading suprascapularis, infrascapularis, subscapularis, and deltoid muscle with necrotic component and lytic lesion in scapula (Figure 3). Multiple enlarged nodes of size 15 × 20 mm in right axillary and supraclavicular region were also found. Computed tomography evaluation of thorax revealed no metastatic lesion in lung parenchyma. Ultrasonography of abdomen was within normal limit. This created confusion whether to treat it as primary rhabdomyosarcoma or metastatic germ cell tumor based on previous history of orchidectomy. Tumor markers, that is, serum AFP, were 21.92 (ng/mL), Beta HCG-72.20 (IU/L), and LDH-5311.2 (IU/L). Immunohistochemistry revealed that vimentin, desmin, and CD99 were negative which excluded the possibilities of sarcoma. But it was positive for cytokeratin. Based on histopathology, raised tumor markers, and immunohistochemistry the scapular swelling was diagnosed as metastatic nonseminomatous germ cell tumor of previously orchidectomised right testicular tumor. Patient was treated with chemotherapy BEP regimen having bleomycin 18 IU/m^2^ on D_1_, D_8_, and D_15_, etoposide 100 mg/m^2^, and cisplatinum 20 mg/m^2^ of D_1_–D_5_ at 3-week interval of total 4 cycles, followed by 1 cycle of EP (etoposide and cisplatinum). He had complete response of the scapular lesion (Figure 4) and markers *β*-hCG, *α*-fetoprotein, and LDH were normal after completion of chemotherapy. He was subsequently treated with external beam radiotherapy to the scapula of total 40 Gy in 20 fractions. Patient was advised for regular follow-up at 2-month interval for the first year, 3-month interval for the 2nd year, and 6-month interval for the 3rd to 5th year. At every follow-up tumor marker and at 6-month interval computed tomographic evaluation of thorax and abdomen was advised. He had complete response up to 36 months of follow-up.

## 3. Discussion

Primary testicular tumors may originate from germ cells, sex cord cells, or less commonly peritubular stromal and hematopoietic migratory cells [3]. More than 90% of all tumors are of germ cell origin and malignant. For practical clinical purposes these germ cell tumors are classified into two major groups: seminomas and nonseminomatous germ cell tumors (NSGCT). The group of nonseminomatous germ cell tumors comprises several histologic subsets including pathologic entities such as embryonal carcinoma, yolk sac carcinoma, choriocarcinoma, teratoma, and mixed germ cell tumors.

The sites of metastasis in order of descending frequency are lung, retroperitoneal lymph node, liver, mediastinal lymph node, brain, kidney, gastrointestinal tract, bones, adrenal, peritoneum, and spleen [4]. Hematogenous bone metastasis is uncommon in germ cell tumor.

In a retrospective study of 297 patients with metastatic testicular and extragonadal germ cell tumor, Hitchins et al. reported that incidence of bone metastasis was 3% at presentation and 9% at relapse [5]. Synchronous metastasis commonly involving lung and paraaortic node was present in all patients with bone metastasis. In the present study patient had isolated scapular bone metastasis without involving other organs.

In another retrospective study of 2550 cases of germ cell tumor Jamal-Hanjani et al. observed that most cases of bone metastasis (58%) were of nonseminomatous histopathology and serum tumor markers were elevated in 89% cases [6]. The reported patient was a case of nonseminomatous germ cell tumor of right testis, with scapular metastasis and raised tumor marker.

Vertebra particularly lumbar vertebra (79%) is the commonest site of involvement followed by pelvis (16%), ribs (16%), and femur (11%). None of the above two studies reported scapular metastasis. The mean age of bone metastasis was 40 years, but in the present case the patient was a young male of 22 years, with scapular metastasis.

The histologic features of metastatic lesions are usually the same as those of the primary tumor except for seminoma in which metastatic lesions may be of a different histology in one-third of the patients. Chemotherapy may produce significant changes in pathology. These changes are identifiable by routine microscopy but sometimes immunohistochemistry may be required to properly characterize all the cellular components of metastatic tumors [7]. The primary surgeon at peripheral hospital, due to lack of oncology knowledge, did not submit the right testicular mass for histopathological study. Patient presented to us with left scapular mass and absence of right testis was an incidental finding during general examination. Based on this history, general examination, histopathology, immunohistochemistry, and raised tumor markers this tumor was proved to be a case of metastatic nonseminomatous germ cell tumor of right testis with metastasis to scapula.

Although many studies have examined different chemotherapy regimens including high dose chemotherapy, the standard first line therapy remains four cycles of bleomycin, etoposide, and cisplatinum (BEP) [8, 9]. Grommes et al. reported improved survival with platinum based chemotherapy in germ cell tumors with epidural spinal cord involvement [10]. Chargari et al. observed complete response to BEP in testicular germ cell tumor with solitary skull bone metastasis [11]. The reported case had complete response to BEP regimen.

This is a rare case of nonseminomatous germ cell tumor of right testis with scapular metastasis. This study revealed the importance of tumor markers and immunohistochemistry in diagnosis of metastatic germ cell tumors. Platinum based chemotherapy regimen is the standard of care of metastatic germ cell tumor.
